# The Effect of Strontium-Substituted Hydroxyapatite Nanofibrous Matrix on Osteoblast Proliferation and Differentiation

**DOI:** 10.3390/membranes11080624

**Published:** 2021-08-14

**Authors:** Shiao-Wen Tsai, Yu-Wei Hsu, Whei-Lin Pan, Fu-Yin Hsu

**Affiliations:** 1 Graduate Institute of Biomedical Engineering, Chang Gung University, Taoyuan 33302, Taiwan; swtsai@mail.cgu.edu.tw; 2 Department of Periodontics, Chang Gung Memorial Hospital, Taipei 33305, Taiwan; helen481209@gmail.com; 3 Department of Bioscience and Biotechnology, National Taiwan Ocean University, Keelung 20224, Taiwan; qazwest74@gmail.com

**Keywords:** strontium, hydroxyapatite, nanofiber, osteoblast

## Abstract

Natural bone tissue consists primarily of bioapatite and collagen. Synthetic hydroxyapatite (HA) possesses good biocompatibility, bioactivity, and osteoconductivity due to its chemical and biological similarity to bioapatite. Hence, HA has been widely used as a bone graft, cell carrier and drug/gene delivery carrier. Moreover, strontium-substituted hydroxyapatite (SrHA) can enhance osteogenic differentiation and inhibit adipogenic differentiation of mesenchymal stem cells. Hence, SrHA has the potential to be used as a bone graft for bone regeneration. It is widely accepted that cell adhesion and most cellular activities are sensitive to the topography and molecular composition of the matrix. Electrospun polymer or polymer-bioceramic composite nanofibers have been demonstrated to enhance osteoblast differentiation. However, to date, no studies have investigated the effect of nanofibrous bioceramic matrices on osteoblasts. In this study, hydroxyapatite nanofiber (HANF) and strontium-substituted hydroxyapatite nanofiber (SrHANF) matrices were fabricated by electrospinning. The effect of the HANF components on MG63 osteoblast-like cells was evaluated by cell morphology, proliferation, alkaline phosphatase activity (ALP) and gene expression levels of *RUNX2*, *COLI*, *OCN* and *BSP*. The results showed that MG63 osteoblast-like cells exhibited higher ALP and gene expression levels of *RUNX2*, *COLI*, *BSP* and *OCN* on the SrHANF matrix than the HANF matrix. Hence, SrHANFs could enhance the differentiation of MG63 osteoblast-like cells.

## 1. Introduction

The development of multifunctional bioactive materials for repairing large bone defects caused by trauma, infection, skeletal abnormalities or tumor resection is an important issue [1,2,3]. Bone defects caused by infected nonunion make it difficult to eradicate pathogens. Hence, chronic bone infection usually needs long-term systemic antibiotic treatment after surgical debridement. However, the long-term administration of antibiotics could cause antibiotic resistance. Hence, the development of bone grafts that possess the capacity of sustained antibiotic release and the stimulation of bone regeneration for the treatment of bone defects due to osteomyelitis is of great interest [4]. It has been known for years that bioactive ceramics, such as bioglass, α-tricalcium phosphate, β-tricalcium phosphate, tetracalcium phosphate and hydroxyapatite (HA), are extensively applied for hard tissue augmentation and replacement. HA is the primary inorganic component of bone tissue. Therefore, synthetic HA has been broadly used as bone grafts, cell carriers and drug carriers due to its biocompatibility, bioactivity and osteoconductivity properties. Zhang et al. [5] pointed out that Sr-substituted hydroxyapatite (SrHA) obtained by the partial substitution of Ca^2+^ by Sr^2+^ has higher solubility than pure HA. Tsai et al. [6] showed that the substitution of Ca with Sr induced distortions in the HA lattice, causing the dissolution ratio of SrHA to increase with the Sr doping level. Ni et al. [7] found that HA ceramics with 5 and 10 mol% Sr substitution enhanced osteoblastic cell differentiation and mineralization. Sr ions may promote the differentiation of osteoblast cells by activating downstream Cbfa1 [8]. Moreover, Boanini et al. pointed out that SrHA inhibited osteoclastogenesis and osteoclast differentiation by regulating the RANKL/RANK/OPG signaling pathway [9]. Ge et al. [10] fabricated a SrHA/PLLA scaffold and found the ALP activity of the MC3T3-E1 cells in SrHA/PLLA scaffolds was 2- and 7-fold higher than that of the MC3T3-E1 cells in the HA/PLLA and PLLA scaffolds, respectively. In addition, many studies have pointed out that Sr ion can be used as an antibacterial reagent. Ravi et al. [11] found that SrHA exhibited antibacterial activity against *Escherichia coli* and *Staphylococcus aureus*.

The native ECM is a three-dimensional network that simultaneously provides favorable sites for cell growth and an array of biological cues to direct cellular behavior. Electrospinning is readily utilized to fabricate nanofibers. The topological structure of the electrospun matrix can mimic the extracellular matrix and influence cell behaviors, such as attachment, migration, proliferation and differentiation. [12,13] Sundaramurth et al. demonstrated that chitosan-PVA nanofibers possessed better adhesion and proliferation of fibroblast cells on the surface of chitosan-poly (vinyl alcohol) electrospun nanofiber membranes than on chitosan-poly (vinyl alcohol) cast membranes [14]. Lin et al. [15] fabricated a mesoporous bioglass/polycaprolactone (MBG/PCL) nanofibrous composite matrix and demonstrated that the MBG/PCL nanofibrous matrix exhibited excellent bone-forming bioactivity and cellular differentiation activity compared with the PCL nanofibrous matrix. Frohbergh et al. [16] fabricated the electrospun hydroxyapatite-containing chitosan nanofibrous scaffold and found that ALP activity in osteoblastic cells growing on the electrospun hydroxyapatite-containing chitosan nanofibrous scaffold was 2.4-fold higher than on electrospun chitosan nanofibrous scaffold. Guan et al. [17] reported electrospinning of poly (3-hydroxybutyrate) (PHB)-based fibrous scaffolds containing 10 wt% nano-HA that exhibited much better support for the attachment, proliferation and differentiation of rat bone marrow stromal cells (BMSCs) than pure PHB scaffolds.

In most studies, HA or SrHA has been used either in bulk or in powder form. Recently, in our past research, hydroxyapatite nanofibers (HANFs) and strontium-substituted hydroxyapatite nanofibers (SrHANFs) were successfully fabricated by electrospinning, and found to possess excellent drug-loading efficiency and the ability to release antibacterial agents in a sustained manner [6,18]. Tsai et al. [19] pointed to SrHANFs incorporated into PCL to form PCL-SrHANF membranes as cell culture substrates. The PCL-SrHANF membranes showed 1.3- and 43-fold increase in ALP activities and calcium deposition, respectively compared to the PCL membranes. The PCL-SrHANF membranes could be an alternative material for guided bone regeneration. However, to date, there are no studies evaluating the osteogenic activity of HA fibrous matrices on osteoblasts. Therefore, the aim of this work is to assess the cellular behavior of osteoblast-like cells on HANF and SrHANF matrices.

## 2. Materials and Methods

### 2.1. Synthesis of HANF and SrHANF Matrices

Hydroxyapatite nanofibers (HANFs) were fabricated by using electrospinning technology based on our previous study with a slight modification [18]. Briefly, 0.5 g of hexadecyltrimethylammonium bromide (CTAB, Sigma-Aldrich Chemical Company, St. Louis, MO, USA) and 2.99 g of triethyl phosphite (TEP, Merck, Darmstadt, Germany) were dissolved in 5 mL of deionized water and 5 mL of ethanol. Calcium nitrate tetrahydrate (7.0 g, Sigma-Aldrich Chemical Company, St. Louis, MO, USA) was dissolved in 5 mL of absolute ethanol and subsequently slowly added dropwise to the above CTAB/TEP solution, with stirring for 2 h, to form the precursor solution. The precursor solution was tightly capped and placed in an oven at 60 °C for 12 h. Next, 2 g of poly (vinyl pyrrolidone) (PVP, MW = 40,000, Sigma-Aldrich Chemical Company, St. Louis, MO, USA) and 0.5 g of P123 were dissolved in 7 mL of absolute ethanol and then added to 3 mL of the precursor solution with stirring for 30 min to obtain a transparent mixed solution. The mixed solution was put into an electrospinning apparatus (FES-COS, Falco Tech Enterprise Co. Ltd., New Taipei City, Taiwan), and electrospinning was performed on aluminum foil. The parameters of the electrospinning process were as follows: The needle size, spinning voltage potential, spinning flow and distance between the needle and the collector were 18 G, 30 kV, 0.508 mL/h and 25 cm, respectively. The nonwoven nanofiber structures were collected on the collector and then calcined at 800 °C with a heating rate of 2.5 °C per min under a nitrogen atmosphere for 1 h to obtain the HANF matrix.

Strontium-substituted hydroxyapatite nanofibers (SrHANFs) were synthesized according to a previously reported procedure with a slight modification [6]. First, 3.0 g of TEP and 0.5 g of CTAB were added to 5 mL of ethanol aqueous solution (50% *v*/*v*) and stirred until the solution was clear. Calcium nitrate tetrahydrate (4.896 g, Sigma-Aldrich Chemical Company, St. Louis, MO, USA) was dissolved in 4.5 mL of absolute ethanol and 0.5 mL of deionized water. Strontium nitrate (1.876 g) was dissolved in 3 mL of deionized water. Subsequently, the calcium nitrate and strontium nitrate solutions were slowly added dropwise to the CTAB/TEP solution to form a precursor solution, which was then placed in an oven at 60 °C for 12 h. Next, 2.25 g of PVP and 0.675 g of Pluronic P123 were mixed and dissolved in 7 mL of absolute ethanol and then added to 3 mL of the above precursor solution with stirring for 30 min. This mixed solution was put into an electrospinning apparatus to obtain the SrHANF matrix. The electrospinning parameters and calcine treatment conditions were the same as those described above.

### 2.2. Characterization of the HANF and SrHANF Matrices

Scanning electron microscopy (SEM, ZEISS ΣIGMA Essential) was performed to characterize the morphology of the HANF and SrHANF matrices. The average fiber diameters of HANF and SrHANF were determined by applying image analysis software (Image-Pro Express, Media Cybernetics, Rockville, MD, USA) to SEM images. The phase compositions of HANF and SrHANF were characterized by X-ray diffraction (XRD, Bruker D2-Phaser, Madison, WI, USA). The diffraction angle (2θ) was scanned from 20° to 70° with a step size of 0.04°.

### 2.3. Cellular Proliferation on HANF and SrHANF Matrices

The HANF and SrHANF matrices (1 cm × 1 cm) were sterilized by exposure to UV light overnight in a laminar airflow hood prior to cell culture. The sterilized membranes were placed in 24-well tissue culture plates containing a suspension of MG63 osteoblast-like cells (BCRC no. 60279, Bioresource Collection and Research Center, Hsinchu, Taiwan) (1 × 10^4^ cells/well) in minimum essential medium (MEM) supplemented with antibiotics (100 U/mL penicillin and 100 µg/mL streptomycin) and 10% FBS. The cell metabolic activity of MG63 on the HANF and SrHANF matrices was examined using the 3-(4,5-dimethylthiazol-2-yl)-2,5-diphenyltetrazolium bromide (MTT) assay after 1, 3 and 7 days of incubation. The rate of cellular proliferation was calculated according to the following formula:

Rate of cellular proliferation = OD_570_ on day 7/OD_570_ on day 1

### 2.4. Cytoskeleton Organization on HANF and SrHANF Matrices

MG63 cells cultured on the HANF and SrHANF matrices were stained with rhodamine-phalloidin to observe the cytoskeletal organization. First, the matrices were fixed with 3.7 wt% paraformaldehyde in phosphate-buffered saline (PBS, 0.02 M pH = 7.4). Subsequently, the matrices were rinsed with 0.1 wt% Triton X-100 in PBS for 5 min and incubated with 1 wt% bovine serum albumin in PBS for 1 h to block nonspecific background staining. After blocking, the matrices were incubated with Alexa Fluor^®^488 phalloidin to stain the cytoskeleton through the binding of phalloidin to F-actin. Next, the matrices were incubated with 0.1 wt% 2-(4-amidinophenyl)-6-indolecarbamidine (DAPI) in PBS to stain the cell nuclei. Finally, the matrices were rinsed with PBS and observed by laser scanning confocal microscopy (LSCM, Zeiss LSM 780 META, Jena, Germany). The cytoskeleton and nuclei were stained green and blue, respectively.

### 2.5. Alkaline Phosphatase (ALP) Activity of MG63 on HANF and SrHANF Matrices

The osteogenic differentiation of each cell-seeded matrix was evaluated as a function of ALP activity on days 3, 7 and 14. The cell-seeded matrices were washed with PBS, and ALP was extracted by lysing the cells in lysis buffer solution (0.1 M glycine, 1 mM MgCl_2_, and 1 wt% Triton X-100 in PBS) for 20 min. After lysis, 50 μL of cell lysate was incubated with 150 μL of the ALP activity reagent (Randox ALP Detection Kit, Crumlin, UK) for 10 min at 37 °C. The ALP activity was measured at 405 nm using a spectrophotometric microplate reader (Biotek uQuant, Winooski, VT, USA). The protein content of cell lysates was also analyzed using the Pierce BCA Protein Assay Kit (Thermo Scientific, Waltham, MA, USA). The relative ALP activity was calculated by normalizing the ALP activity to the total protein content.

### 2.6. Gene Expression Analysis Using Real-Time Quantitative Polymerase Chain Reaction (Q-PCR)

After 3, 7 and 14 days of culture, total RNA was extracted from the cell-seeded matrices using TRI Reagent. In brief, 1 mL of ice-cold TRI Reagent was added to the matrix and incubated for 5 min at room temperature. Subsequently, 0.1 mL of 1-bromo-3-chloropropane was added and incubated for 15 min. Aliquots of this mixture were centrifuged (12,000× *g* for 15 min at 4 °C), and the upper aqueous phase was collected. The RNA was precipitated from the aqueous phase by mixing with isopropyl alcohol and centrifuging (12,000× *g* for 10 min at 4 °C). Following precipitation, the RNA pellet was washed with 75% ethanol and recentrifuged (7500× *g* for 5 min). The resulting pellet was air-dried and redissolved in diethylpyrocarbonate (DEPC)-treated water. To evaluate the DNA concentration and purity, sample aliquots were measured using a spectrophotometer at an absorbance of 260 nm and the absorbance ratio of 260 and 280 nm.

The osteogenic differentiation of MG63 cells in various matrices was evaluated by measuring the gene expression levels of runt-related transcription factor 2 (*RUNX2*), collagen type I (*COLI*), osteocalcin (*OCN*) and bone sialoprotein (*BSP*) using Q-PCR. The gene expression level of glyceraldehyde 3-phosphate dehydrogenase (*GAPDH*) was used as an internal control. Q-PCR oligonucleotide primer sequences are shown in Table 1. Briefly, 10 µL of SYBR Green PCR Master Mix (Protech SA-SQGLR-V2), 2.5 µL of cDNA, 2.1 µL of ddH_2_O and 0.5 µL each of the forward and reverse primers were mixed. After the initial denaturation at 95 °C for 15 min, the target genes were amplified with 40 cycles of denaturation at 95 °C for 15 s and annealing at 60 °C for 60 s. Real-time PCR was carried out on an iQ5 gradient real-time PCR detection system (Bio-Rad, Hercules, CA, USA). The relative gene expression level was calculated by the 2^−∆∆Ct^ method. MG63 cells cultured in cell culture plates were used as control groups. The fold changes were calculated using the following formulas:

SampleΔC_t_ = C_t sample_ − C_t *GAPDH*_

ΔΔC_t_ = SampleΔC_t_ − controlΔC_t_

The fold change of the sample vs. the control = 2^−∆∆Ct^

### 2.7. Statistical Analyses

Statistical analyses were performed using SPSS v.10. Fiber diameters were analyzed with Student’s t test. Cellular metabolic activity, ALP activity and gene expression were analyzed with the nonparametric Mann–Whitney U test. A *p*-value < 0.05 was considered statistically significant.

## 3. Results and Discussion

### 3.1. Characterization of HANF and SrHANF Matrices

**Figure 1:**
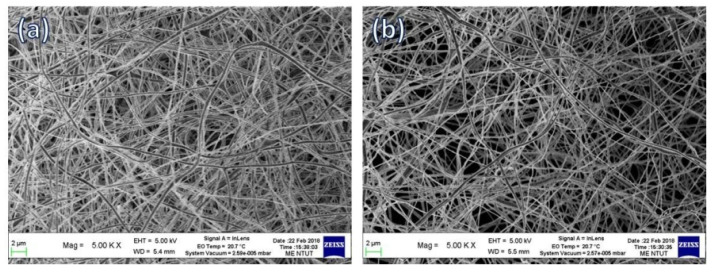
Microstructure of matrix. SEM images of (**a**) SrHANF and (**b**) HANF.

Figure 1 shows the SEM images of the HANF and SrHANF matrices. The average diameters of HANF and SrHANF were 287 ± 61 and 258 ± 74 nm, respectively. The nanofiber diameter was not significantly different between HANF and SrHANF (*p* > 0.05). The microstructure and fiber diameter of HANF and SrHANF matrices mimic the extracellular matrix (ECM).

**Figure 2:**
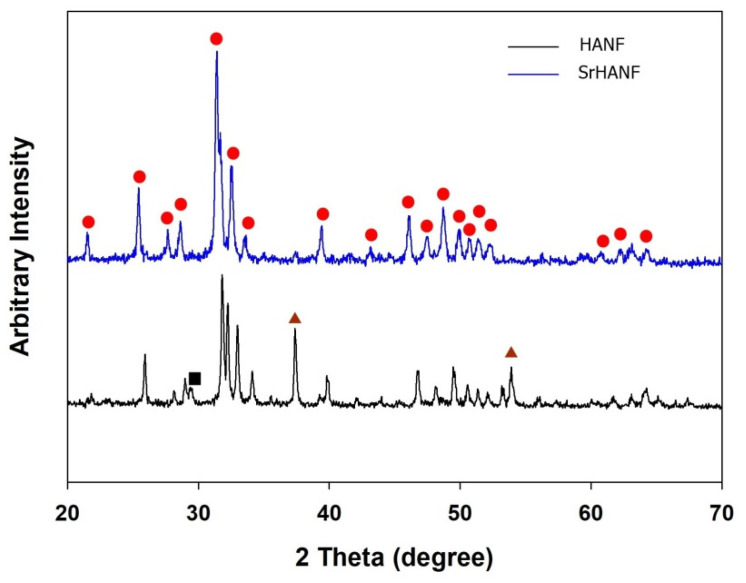
Determination of crystallographic phases of matrix. XRD patterns of SrHANF and HANF. (▲: CaO; ●: HA/SrHA; ■: CaCO_3_).

The crystallographic phases of the HANF and SrHANF matrices were studied by wide-angle X-ray diffraction analysis. The XRD patterns of the HANF and SrHANF matrices are shown in Figure 2. The HANF matrix showed several peaks at 2θ = 21.8°, 25.8°, 31.7°, 32.9°, 39.9°, 46.7° and 49.5° corresponding to HA (PDF card: #89-5632), peaks at 2θ = 32.0° and 37.6° corresponding to calcium oxide (PDF card #01-1160), and peaks at 2θ = 29.6° corresponding to calcium carbonate (PDF card #85-1108). In contrast, the SrHANF matrix showed several peaks at 2θ = 21.7°, 25.6°, 31.5°, 31.8°, 32.7°, 39.5°, 46.2° and 48.9° corresponding to strontium-substituted hydroxyapatite (SrHA). In addition, only small amounts of calcium oxide and calcium carbonate were present in the SrHANF matrix. The characteristic diffraction peaks of HA on the SrHANF matrix were shifted to lower 2θ values than the HANF matrix, which indicates that the d-spacings increased upon Sr incorporation because the atomic radius of Sr (118 pm) is larger than that of Ca (100 pm) [20].

**Figure 3:**
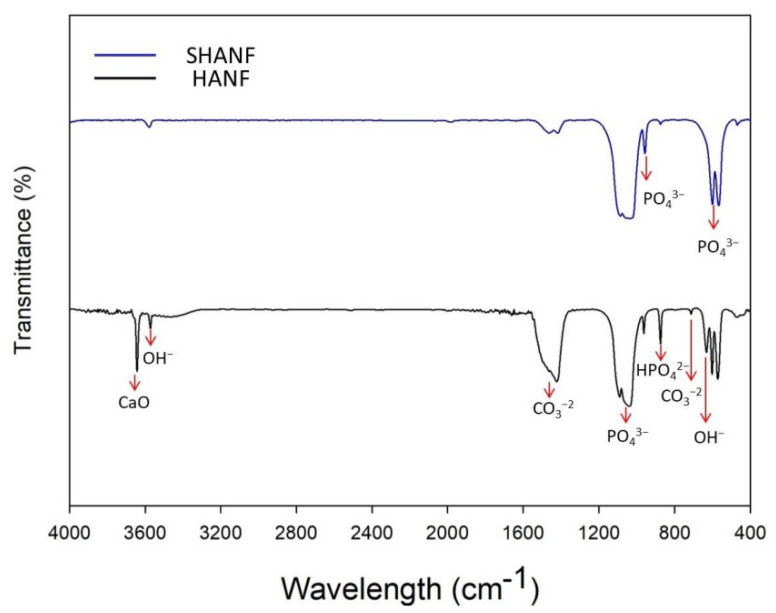
Determination of the infrared-absorption peaks of the matrix. Fourier transform infrared spectroscopy (FTIR) spectra of SrHANF and HANF.

Figure 3 shows the characteristic FTIR spectrum for the SrHANF and HANF. The characteristic peaks were observed at approximately 566 and 609 cm^−1^ (P-O bending) and at 960 and 1000~1100 cm^−1^ (P-O stretching) in PO_4_^3−^. Moreover, the characteristic peaks were observed at approximately 710 and 1440~1470 cm^−1^ are attributable to the CO_3_^−2^ group. The characteristic peak at 875 cm^−1^ was assigned to the HPO_4_^2−^ group in HA. The characteristic peaks were observed at 632 (OH^−^ stretching) and 3571 cm^−1^ (OH^−^ bending vibration) in HA. Additionally, the sharp peak at 3642 cm^−1^ was observed in the spectrum of HANF, confirming the formation of the CaO phase [18].

### 3.2. Cellular Behavior on the HANF and SrHANF Matrices

**Figure 4:**
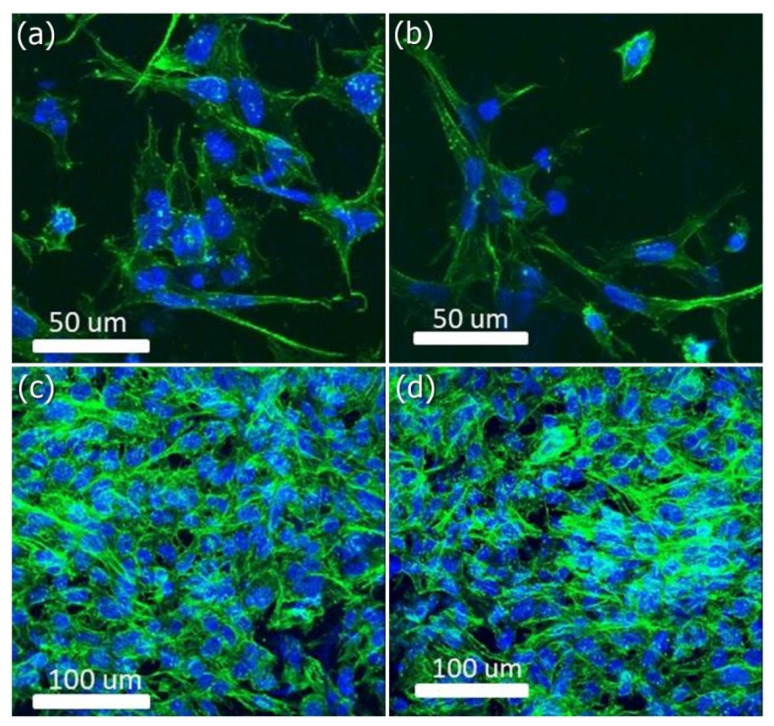
Cellular morphology of MG63 osteoblast-like cells on matrix. Representative images of MG63 osteoblast-like cells cultured on SrHANF and HANF. (**a**,**c**) SrHANF and (**b**,**d**) HANF; (**a**,**b**) after one day of culture. (**c**,**d**) after four days of culture. Cytoskeletal F-actin was stained green with FITC, and cell nuclei were stained blue with DAPI.

FITC-conjugated phalloidin was used to visualize the F-actin cytoskeleton of MG-63 cells on the HANF and SrHANF matrices under LSCM (as shown in Figure 4). Spindle-shaped cellular morphology was observed on the HANF and SrHANF matrices two days after cell seeding (as shown in Figure 4a,b). Moreover, MG63 cells grew and exhibited well-spread stellate-shaped cellular morphology on the HANF and SrHANF matrices after four days of culture (Figure 4c,d). Hence, the HANF and SrHANF matrices showed excellent biocompatibility.

**Figure 5:**
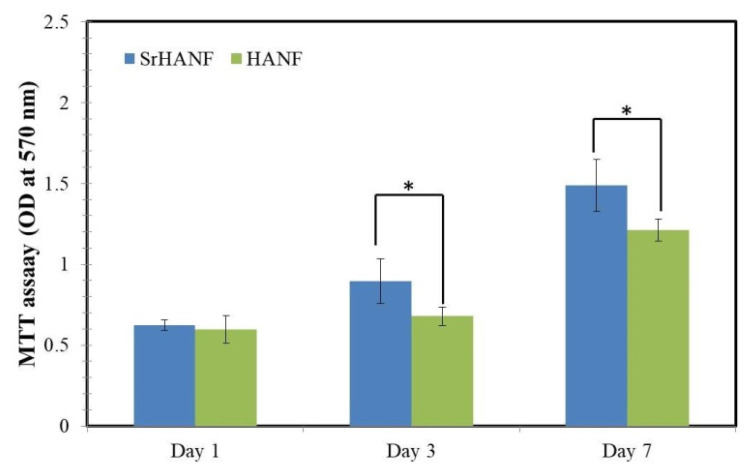
Quantification of MG63 osteoblast-like cells on SrHANF and HANF matrices. MTT assay quantifying cell proliferation on SrHANF and HANF. The viability of MG63 osteoblast-like cells on various matrices after culture for up to seven days. Data are presented as the mean ± SD, *n* = 3. Statistical analysis comparing SrHANF and HANF. (*) Denotes a significant difference (*p* < 0.05).

Cell growth is sensitive to the composition of the matrix. The proliferation rate of MG63 cells on the HANF and SrHANF matrices were analyzed using MTT assays. The MG63 cells incubated on the SrHANF matrix showed significantly higher cellular proliferation rate (2.39) than those incubated on the HANF matrix (2.02) after seven days of cell culture (as shown in Figure 5).

**Figure 6:**
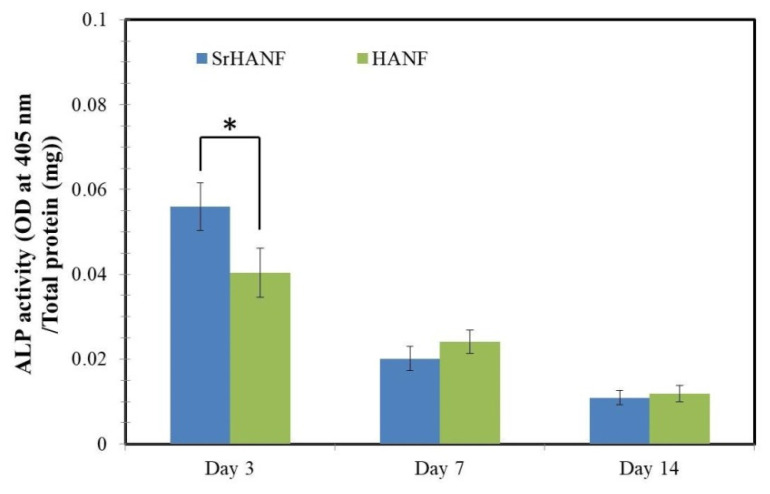
Analysis of alkaline phosphatase activity of cells on SrHANF and HANF matrices. Relative ALP activity of MG63 osteoblast-like cells on SrHANF and HANF after seven days of incubation. The data are presented as the mean ± SD, *n* = 3. Statistical analysis comparing SrHANF and HANF. (*) Denotes a significant difference (*p* < 0.05).

ALP activity is widely recognized as a relatively early biochemical marker for osteoblast differentiation. The phenotype of MG63 cells cultured on the HANF and SrHANF matrices was investigated by analyzing their ALP activity after up to 14 days of culture, as shown in Figure 6. On the third day, the SrHANF matrix has the highest ALP activity, which is 1.4-fold higher than that of the HANF matrix (*p* < 0.05).

**Figure 7:**
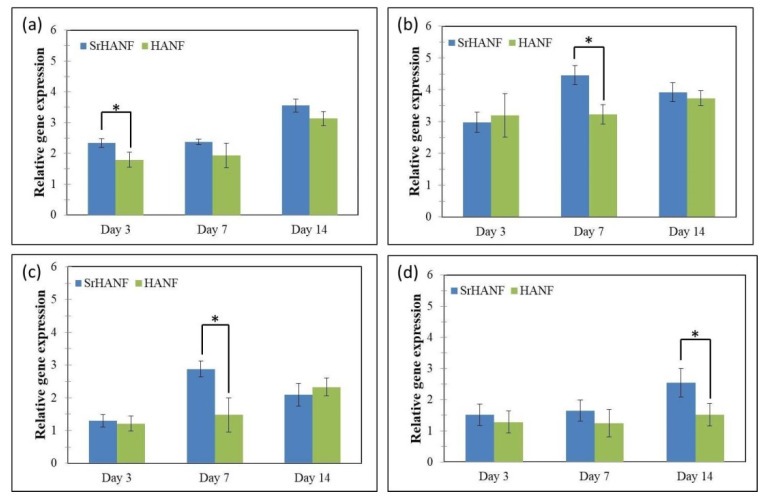
Matrices regulate the bone-associated genes expressed by MG63 osteoblast-like cells. Real–time PCR analyses of bone-associated genes expressed by MG63 osteoblast-like cells on SrHANF and HANF: (**a**) *COLI*, (**b**) *RUNX2*, (**c**) *BSP* and (**d**) *OCN* levels after normalization to *GAPDH*. Data are shown as the fold change relative to the petri dish after three days of incubation. Statistical analysis comparing SrHANF and HANF. (*) Denotes a significant difference (*p* < 0.05).

The expression levels of MG63 osteoblast-specific genes were analyzed using Q-PCR for *COLI*, *RUNX2*, *OCN* and *BSP*, as shown in Figure 7. *COLI* is a specific early-stage marker of osteoblast differentiation during the proliferative and matrix maturation phases [21]. The collagen gene expression of MG63 cells was significantly higher on the SrHANF matrix than on the HANF matrix on day three, which suggested its high proliferation and differentiation (Figure 7a).

*RUNX2* is an essential transcription factor for the initiation of osteogenesis. The *RUNX2* gene expression of MG63 cells was significantly higher in the SrHANF and HANF matrices than in the control group. Moreover, MG63 cells cultured on the SrHANF matrix exhibited significantly higher *RUNX2* gene expression on day seven than cells cultured on the HANF matrix (Figure 7b). Peng et al. showed that strontium could greatly increase *RUNX2* transcriptional activity and phosphorylation [22]. Aimaiti pointed out that strontium could enhance osteogenesis and matrix mineralization via the ERK signaling pathway [23]. ERK signaling pathways can activate the transcription of *RUNX2* during osteoblast differentiation [24].

The gene expression of *BSP* was regulated by *RUNX2* in the osteoblast differentiation process [25]. *BSP* is a phosphoprotein that is normally expressed in abundance by osteoblasts and can bind to HA via its HA binding site. The precise biological function of *BSP* is still unknown. Some studies have found that BSP expression has a direct effect on osteoblast differentiation and can promote the initial formation of mineral crystals [26]. Hence, *BSP* is considered a middle-stage marker of osteoblastic differentiation. In comparison to MG63 cells grown on HANF matrix, MG63 cells grown on the SrHANF matrix showed significantly higher gene expression of *BSP* at seven days (Figure 7c).

*OCN* is the target gene of *RUNX2* and is regulated by *RUNX2* [27]. The increase in *OCN* gene expression was evidence of the maturation of osteoblasts. *OCN* is considered a late-stage marker of osteogenic differentiation. The results showed that the *OCN* gene expression of MG63 cells was significantly higher on the SrHANF matrix than on the HANF matrix on day 14 (Figure 7d).

## 4. Conclusions

Osteoblast-like MG63 cells grown on SrHANF matrix showed higher levels of *COLI*, *RUNX2*, *BSP*, and *OCN* mRNA and higher ALP activity than those grown on HANF matrix. The results indicated that the SrHANF matrix enhanced osteoblast differentiation compared to the HANF matrix. Hence, the SrHANF is a highly promising potential bone graft for guided bone regeneration in bone defect.

## Figures and Tables

**Table 1:** Oligonucleotide primer for PCR amplification.

Gene	Primer Sequence: Sense/Antisense
*GAPDH*	5′-GAGTCCACTGGCGTCTTCACC-3′
	5′-GACTGTGGTCATGAGTCCTTC-3′
*RUNX2*	5′-GGAGGGACTATGGCATCAAA-3′
	5′-GCTCGGATCCCAAAAGAAGT-3′
*COLI*	5′-CGGAGGAGAGTCAGGAAG-3′
	5′-CAGCAACACAGTTACACAAG-3′
*BSP*	5′-TGCCTTGAGCCTGCTTCCT-3′
	5′-CTGAGCAAAATTAAAGCAGTCTTCA-3′
*OCN*	5′-CAGCGAGGTAGTGAAGAC-3′
	5′-GCCAACTCGTCACAGTCC-3′

## Data Availability

Not applicable.
